# Offshore Evidence for an Undocumented Tsunami Event in the ‘Low Risk’ Gulf of Aqaba-Eilat, Northern Red Sea

**DOI:** 10.1371/journal.pone.0145802

**Published:** 2016-01-27

**Authors:** Beverly Goodman Tchernov, Timor Katz, Yonathan Shaked, Nairooz Qupty, Mor Kanari, Tina Niemi, Amotz Agnon

**Affiliations:** 1 University of Haifa, Mt. Carmel, Israel; 2 Israel Oceanographic and Limnological Research, Shikmona, Israel; 3 Interuniversity Institute of Marine Sciences-Eilat, Coral Beach, Israel; 4 Tel Aviv University, Tel Aviv, Israel; 5 University of Kansas-Missouri, Kansas City, Missouri, United States of America; 6 Hebrew University of Jerusalem, Jerusalem, Israel; Institute of Tibetan Plateau Research, CHINA

## Abstract

Although the Gulf of Aqaba-Eilat is located in the tectonically active northern Red Sea, it has been described as low-risk with regard to tsunami activity because there are no modern records of damaging tsunami events and only one tsunami (1068 AD) referred to in historical records. However, this assessment may be poorly informed given that the area was formed by and is located along the seismically active Dead Sea Fault, its population is known to fluctuate in size and literacy in part due to its harsh hyper-arid climate, and there is a dearth of field studies addressing the presence or absence of tsunamigenic deposits. Here we show evidence from two offshore cores for a major paleotsunami that occurred ~2300 years ago with a sedimentological footprint that far exceeds the scarce markers of the historically mentioned 1068 AD event. The interpretation is based on the presence of a laterally continuous and synchronous, anomalous sedimentological deposit that includes allochtonous inclusions and unique structural characteristics. Based on sedimentological parameters, these deposits could not be accounted for by other transport events, or other known background sedimentological processes.

## Introduction and Background

**Fig 1:**
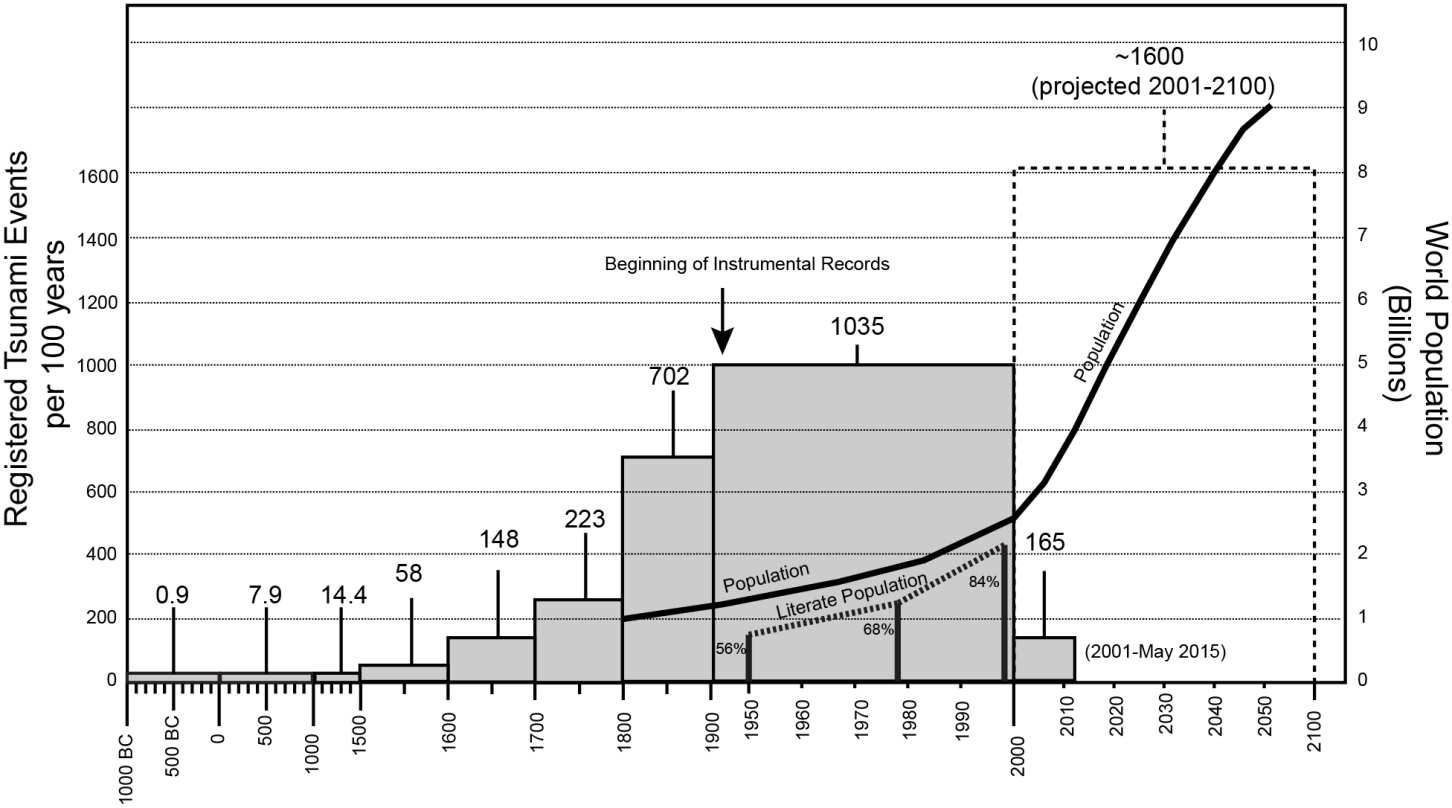
Worlwide tsunami records from past 3000 years. Comparison between number of tsunamis per century recorded [4], world population growth [2], and worldwide literacy rates beginning in 1950 [3].

Written records of past tsunami events only exist in locations where there was a population to witness the event, the means to record it (writing/oral), and the resulting documentation was not destroyed, lost, or forgotten over time. Events that occur in the absence of writing, or are not recorded, are referred to as ‘paleotsunamis’ and are usually discovered by way of field research [1]. A set of relatively recent global circumstances have led to an overall increasing trend in the number of tsunamis recorded each decade. First, population has increased in the past two millennia (minimum 20-fold increase over 2000 years, with an especially pronounced increase in the past 200 years[2] resulting in more people available to witness events as well as being affected by them. Also, technologically, the means of observing, recording, and archiving information has also advanced and the overall global literacy rates have increased [3]. In addition to this, some coastlines that could only support small populations comfortably are now inhabitable due to air conditioning, water desalinization and transport, and other modern conveniences. Consequently, a general correlation exists between the number of tsunamis recorded and how much time has passed [4] (Fig 1). Therefore, the older the event and the fewer the population, the lower the chances are for a written record to exist. Tsunami catalogues are primarily compiled from those records.

The harsh climate (hyperarid desert) of the Gulf of Eilat-Aqaba (GOA) has limited the overall population size of the region, and while there were some phases of more developed harbor towns (Aila, Etzion Gaber) along the ancient trade routes, there were also periods of hundreds of years in which the population was severely limited to small, often seasonal, fishing villages [5]. The single reference to a tsunami in this region is from 1068 AD [6]. In 1950, the combined resident population of the entire Gulf of Eilat-Aqaba (GOA) was less than 15,000, while today it exceeds 200,000 and is higher during the tourist season.

Coastal management and tsunami risk assessments rely on data from tsunami catalogues. Tsunami catalogues aim to centralize all the information known from a defined geographical region, including instrumentally recorded data (limited to recent 100 years), historical records, and field studies. Unfortunately, even given best efforts, this leads to a somewhat inconsistent and geographically patchy collection that does not provide insight to important localized effects and unrecorded events [7]. The recent rise in tsunami sediment field studies [8], both modern and ancient, has increased our understanding of their appearance and improved our ability to recognize the wide range of characteristics that they present in the field [1]. Because of the complexities of positively identifying tsunamigenic sedimentary deposits, the approach is dominated by demonstrating their uniqueness relative to alternative explanations (e.g. floods, large storms, turbidites). This is accomplished by identifying the contrasting features of deposits relative to modern analogues, or inference from intra and interstudy variation [9][10][11][12] though there is ample discussion regarding whether this challenge is sufficiently met [13].

Because of the lack of written tsunami records, for the Red Sea generally, they are not considered a major geohazard [14]. However, given extensive neotectonic and paleoseismic activity along the Dead Sea fault system (over 1400 events earthquakes 1985–1995) [15][16] tsunamis such as the aforementioned 1068 AD event, might be expected. The disparity between earthquake activity and recorded tsunamis may be the result of an actual absence of events, or, it could be the result of inequities in the written record.

With the lack of written records, field research is a critical resource for reconstructing past events in the GOA. Shaked et al. [17] identified onshore evidence of a rapidly buried coral reef that may have been caused by an event associated with an earthquake and marginal faulting. Depositional sequences along the Sinai Peninsula were proposed as possibly tsunamigenic, but never analysed nor assessed for age [18]. In 1995, following a 7.1 Mw earthquake with an epicenter about 80km south of Eilat (28.76 N 34.66 E), a small tsunami was recorded at Nuweiba, Egypt [19][20], confirming some of the suspicions of the area’s tsunami potential. Indisputable coastal tsunami deposits have very poor preservation potential in the region and are unlikely to remain intact and recoverable because of disturbances such as mechanical erosion and anthropogenic interference [21]. Exceptions include protected coastal lakes, lagoons and marshes. The offshore record, in contrast, has been proposed and in some cases proven to have greater preservation potential [22] [23][24][25][26][27].

**Fig 2:**
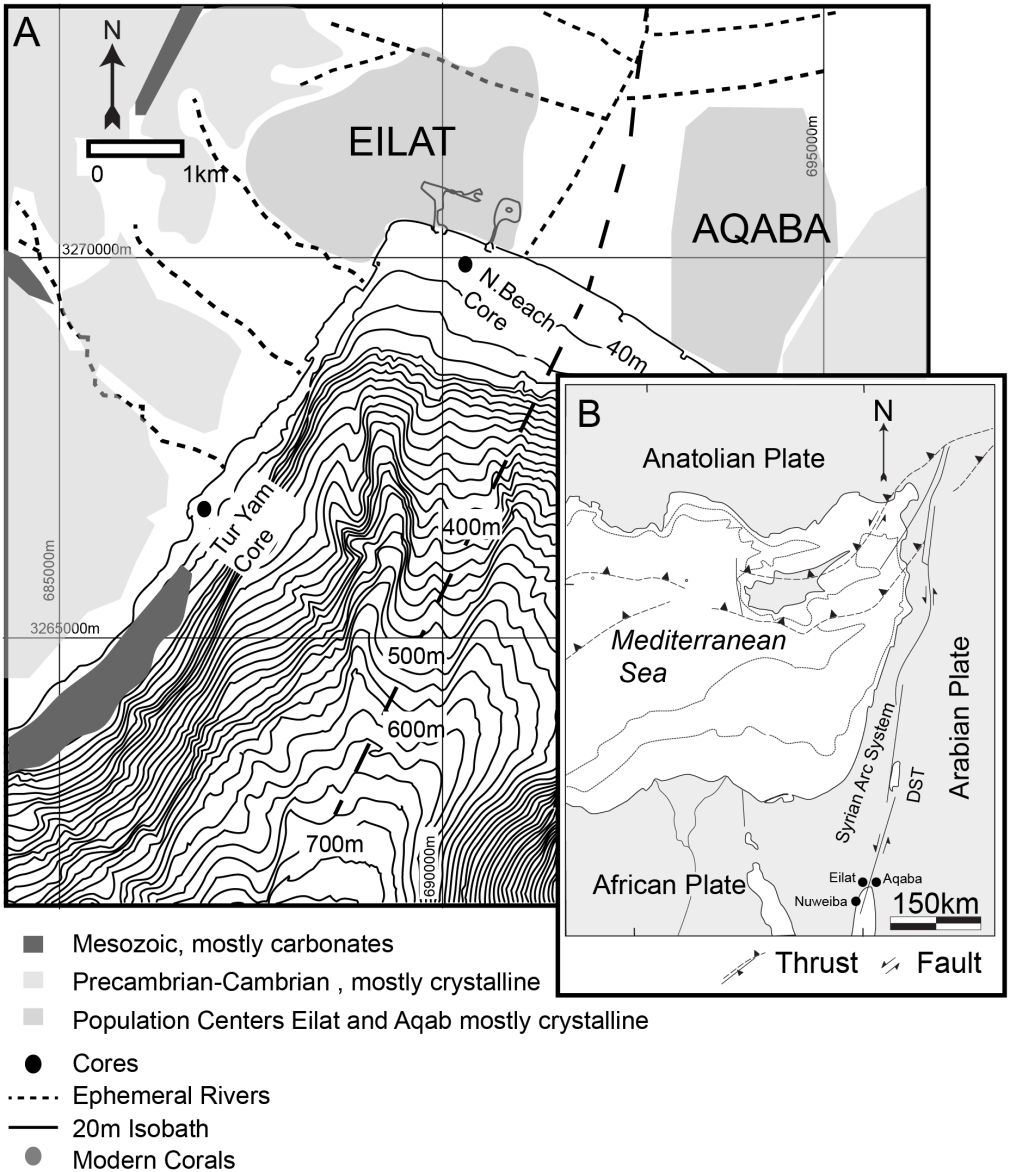
Maps of research site and surrounding areas. a) Site Map (adapted from Tibor et al., 2010), b) Regional Map (adapted from Freund et al., 1970, Garfunkel et al., 1981; Ben-Avraham, Z., 1985).’DST’ = Dead Sea Transform System.

The GOA’s upper shelf zone is variable in sediment character. It is located at the southern part of the Dead Sea Fault System, and is flanked east and west by silicic magmatic and metamorphic mountains with an approximate 105 km left-lateral offset [28][29][30], and a valley stretching north into the flat plains of the Arava desert [31] (Fig 2). The western and eastern flanks are narrow and steep, supporting tropical coral reefs. These sediments are a coarse blend of reef debris and mixed clastic sands and gravels from the nearby mountains. The northern area exhibits a more moderate bathymetry and larger shelf with extensive overlying sediment beds relative to the eastern and western slopes, and does not support coral reefs (Fig 2). The sediments are derived primarily from infrequent flash floods [32]. Northern winds dominate most of the year, resulting in calm conditions, though annual southern storms typically have relatively small wave heights (maximum 3 meters) and only rework sediments at shallow depths (<5m) [33].

In this study, offshore shallow shelf cores were studied to test whether the sedimentological regime has been consistent and homogenous over the past few thousand years, as would be expected in a landscape at low-risk for tsunami events, or if there is any evidence for rapid changes that might suggest otherwise. Our research targeted less disturbed offshore sediments in an attempt to correctly assess possible tsunami risks present in this rapidly growing region.

## Methods

**Fig 3:**
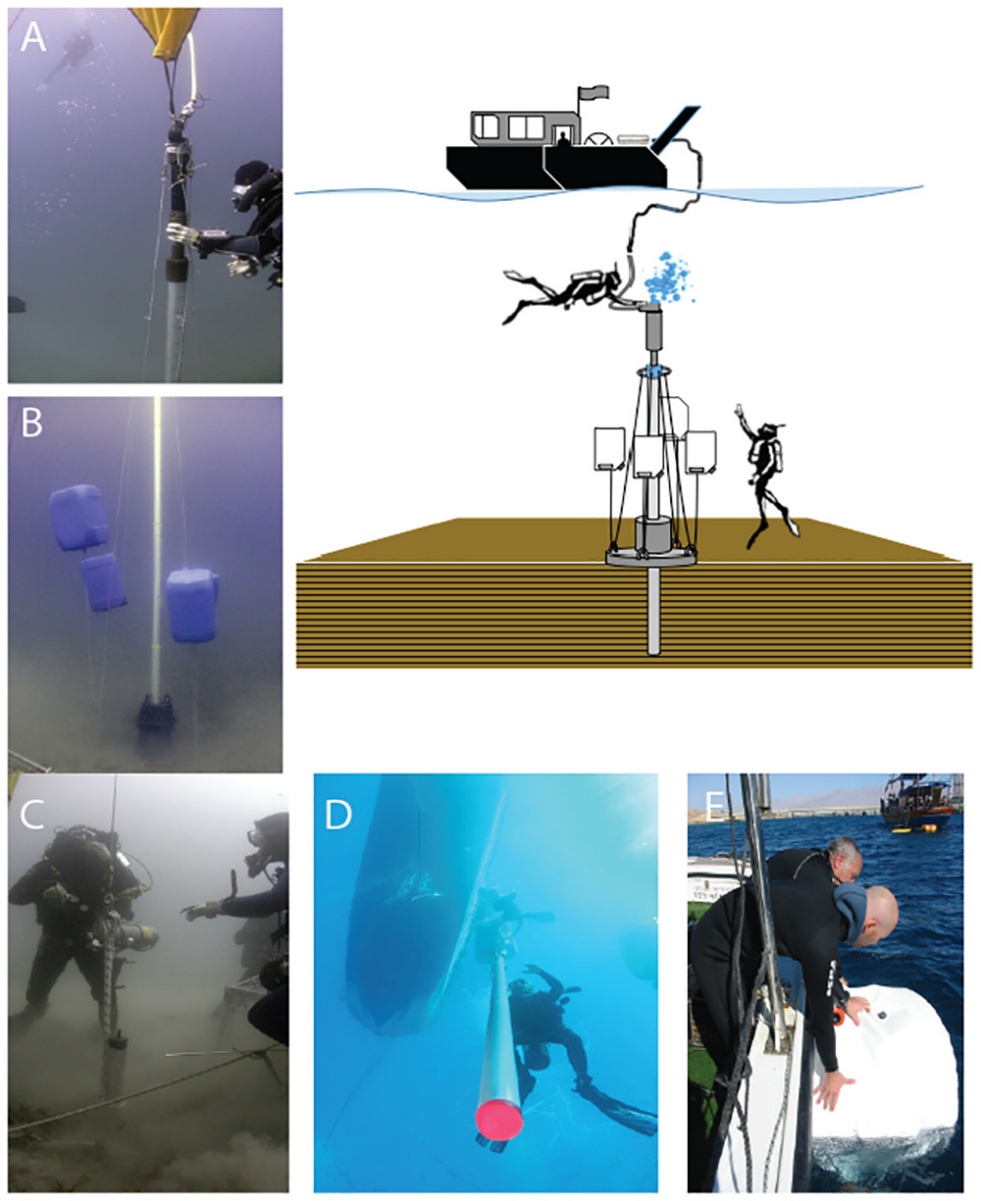
Sandy uppershelf sandy sediment core collection method. A) air hammer, B) weighted base c) preparing core for removal, d) core raised and en route to boat, e) airbag being retrieved prior to raising the sediment core which is attached below. Schematic of system (adapted from Goodman et al. 2009) Photo credits Eran Brokovich, B. Goodman, S. Breitstein.

Cores were collected in two distinctive zones of the GOA (Fig 2); the North Beach, offshore from the discharge of the Wadi Arava (Arava Drainage); and Tur Yam, a small bay offshore from the discharge of Wadi Shlomo (Solomon Drainage). Both sites are outside the coral reef and not within protected marine areas. Divers collected the cores using an adapted pneumatic hammer attached to an aluminium pipe with a onion-style core catcher at the penetration end, which was then secured and counter-balanced with floats at four points (Fig 3). A compressor at the surface provided the hammer’s air supply. At each site, two cores were collected within 75 meters of one another, the longest core (‘primary core’) was selected for analysis and the remaining one was archived. Primary core analyses included sedimentological description, photography, granulometry and micropaleontology. Chronology was established based on C14 dates of marine shell, coral, and foraminifera, selected for least signs of diagenesis, and calibrated using Calib Rev 6.0.

The cores were split lengthwise, described and photographed. Initial descriptions summarized visually documentable characteristics such as sorting, color, shell content, and largest inclusion size. For grain size analysis, subsamples were taken at 1 cm resolution except where inclusions prevented such high-resolution sampling, in which case the minimum possible sample resolution was taken. Hydrogen peroxide (35%) was added to each sample to digest organic matter prior to analysis. Samples were measured with a Beckman LS 320 to determine particle distribution by volume below 2000 micrometers. Results from all measurements were plotted in a three-axis contour map (grain size, percentage abundance, depth in core) using ODV ver. 4.3.10. This method assists at visualizing the concentration of grain size ranges and emphasizes sorting (standard deviation), the nearest proxy to particle size distribution. Subsamples (1.25cc) for micropaleontology were taken from significant horizons based on the results of the sedimentological description and grain size analysis. Each aliquot was sieved at 500 and 125 microns and dried at 60°C. Individual number of foraminifera in each size fraction were counted. Greater than 500 micron fraction was assessed for the presence of discoloring (yellow/black), breakage, and erosion.

## Results

### Tur Yam Core

**Fig 4:**
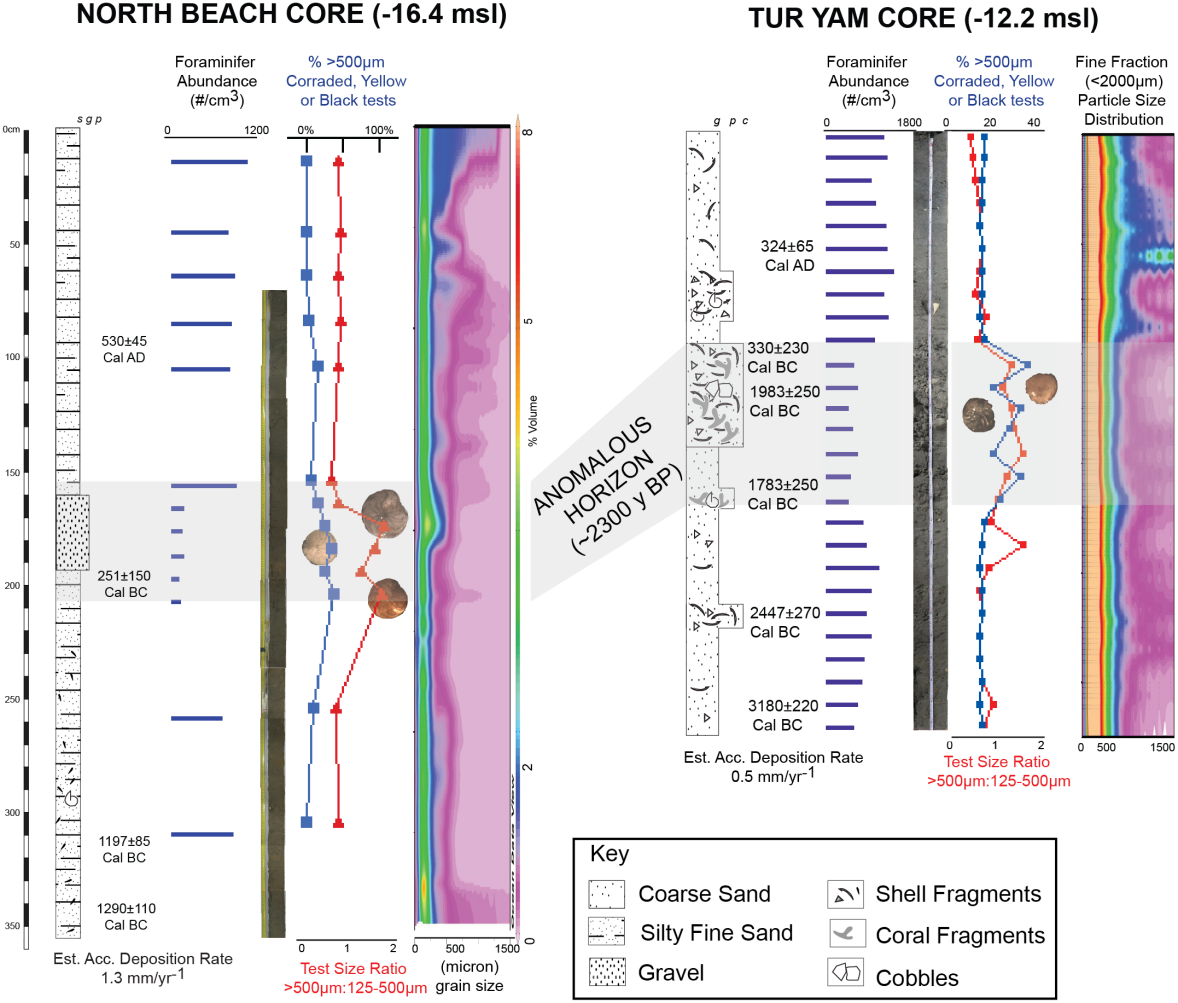
Description and summary of analysis from Tur Yam and North Beach cores. ‘g’ = granule (2-4mm), ‘p’ = pebble (4-64mm), ‘c’ = cobble (64-256mm). Granulometry particle size distribution completed using Ocean Data View version 4.3.10. Correlation between the anomalous horizons of both cores presented. Detail of foraminifer counts and radiocarbon ages available in data repository. Examples of color ranges and corrasion of foraminifera in anomalous horizon as presented in *Amphistegina lobifera* (d’Orbigny 1826).

The Tur Yam core (-12.2 msl, 375cm; Fig 4) is generally characterized by medium sand with fine shell fragments and rare, small (<0.5cm) worn coral fragments, and a mixed mineralogical origin similar to that found in the diverse lithology of the nearby mountains [31] (e.g. metamorphic, magmatic, mixed conglomerates). Midway down core, there is an anomalous bed (~60cm) of more concentrated mixed shell and broken coral fragments of varying condition from pristine to heavily worn and eroded. Ages (Tables A and C in S1 File) within this horizon included 330 ± 230 Cal BC (pristine foraminifera), 1983±250 Cal BC (coral fragment), and 1783±250 Cal BC (coral fragment). Also, the ratio of very large (500+μm) to large (125–500 μm) foraminifera is greater than that of the remainder of the core, while the overall abundance of foraminifera does not change (Fig 2, Table E in S1 File). Eroded, yellowed, or blackened tests (>500 μm) are present in proportions ranging from 14–28%, while the remainder of the core is within the range of 0–13%, with a single exception at 180 cm downcore (~28%). An estimated accumulative deposition rate (0.5 mm yr^-^1) was calculated based on the age from the base of the core and was then adjusted for compaction.

### North Beach Core

The upper portion of the North Beach core (-16.2msl, 450cm; Fig 4) consisted of very fine to fine sand (~75–250 micron mean), foraminifera abundances (>125 micron) in the range of 842–1142 individuals per cm^3^, and a mixed lithology including fine mica, limestone, and granite with a few shell fragments greater than 0.5 cm. A small grain size peak is present at approximately 40 cm downcore (Fig 4). At a depth of 160 cm downcore, the grain size increases to greater than coarse sand (>250 micron) and foraminifer abundances decrease significantly to either low or barren (0–150 individuals per cm^3^). Radiocarbon dating performed on pristine foraminifera and shell within the anomalous band gave an age of 230±35 Cal BC (Tables A and B in S1 File). Of the limited foraminifera present in the anomalous horizon, the proportion of heavily eroded, blackened, or yellowed tests (>500 μm) ranged from 20–31% (Fig 4, Table D in S1 File). The sediment accumulation rate was approximately 1.3 mm/yr^-1^.

## Discussion

A laterally extensive, contemporaneous anomalous horizon (upper radiocarbon age 100–300 years BC) is present across the two study sites (Fig 4). The sediments preceding and following the anomalous layers in both cores resemble the modern surface processes of their respective locations. The sediments from the anomalous layers, in contrast, are more typical of higher energy transport events. The following discussion will consider the likelihood of a range of explanations for this horizon.

### Flooding, Sea-level change, tectonics, storms, and slope failure

**Fig 5:**
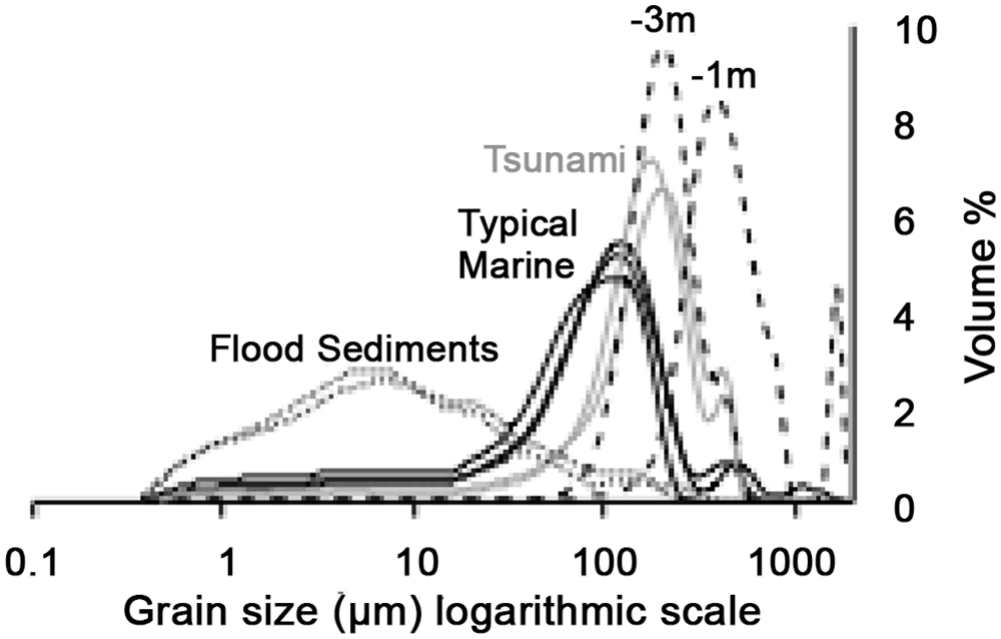
Comparison of North Beach sediment grain size distributions. Dotted black lines are measurements from recent flood sediments. Black dashed lines are modern North Beach seafloor sediments (1m and 3m water depth). Samples from the North Beach core include a set from the anomalous horizon (grey lines) and typical marine background (black lines).

**Fig 6:**
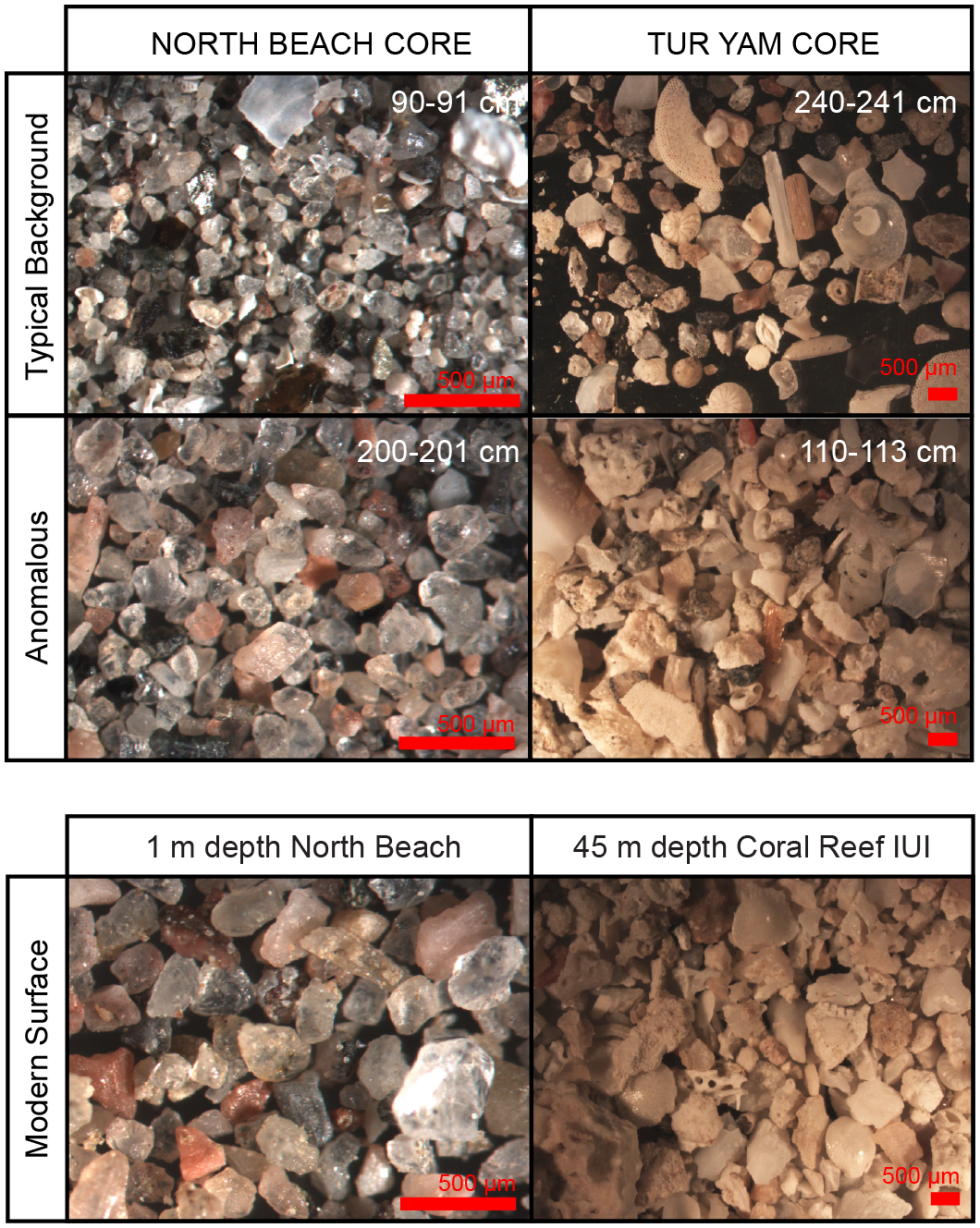
Images of sediments. Photographs of the anomalous and non-anomolous samples from each core and comparative sample from modern collections that bears some resemblance to the anomalous horizon at each site. Upper photos represent typical background samples, which also resemble the modern surface; left: >63 micron sieved sample from 90-91cm North Beach core; right: >500 micron sieved sample from 240–241 cm in Tur Yam core. Middle photographs are examples from the anomalous sections of each core; left >63 micron sieved sample from 200–201 cm North Beach core; right >500 micron sieved sample from 110–113 cm in the Tur Yam core.

In the North Beach core the anomalous horizon is characterized by coarser rather than *finer* sediments relative to the typical background material, demonstrating that they are most likely not the result of a flashflood, which deposit a larger proportion of fine material compared to background sediments [32] (Fig 5). Within the typical background sediments, floodless periods (droughts) are associated with coarser sediments, though not as coarse as those within the anomalous horizon (Fig 6). Also, while drought periods may result in lower sedimentation rates, they do not limit the growth of foraminifera, and might even enhance it, as is demonstrated through the continued high abundance of foraminifers throughout those horizons. The anomalous horizon contains fewer or no foraminifers relative to all other background sediments. At Tur Yam, rare flashfloods can enter, though the narrow shelf and steep bathymetry causes the fine sediments to be quickly removed downslope with little residence time at the depths of the collections. The Tur Yam deposit also contains ample foraminifer and coral debris, which are indicative of a marine rather than terrestrial source.

Because the sediments in the North Beach core are coarser, which is also true of the more nearshore and shallower environment, it might be suggested that either sea-level was lower, or tectonic displacement altered the position relative to sea level. Broadly speaking, beach sediments are coarser than those of greater depths, and sediment size tends to become finer; a result of winnowing and transport processes from source to sink. Therefore, earlier sea level stands could lead to buried beach zones that would appear coarser and with lower foraminifer abundances when sampled, as is seen here. However, sea-level curves from the age of the anomalous horizon vary by no more than two meters [34][35] which would not place the horizon within the equivalent shallow or near shore depths.

Alternatively, tectonics could be considered. A minimum 15 meter vertical tectonic displacement would be necessary to reposition the anomalous horizon, albeit for only a period of time before being ‘re-displaced’ back to its original depth position, again an unlikely explanation, and contradicted by the general evidence for overall vertical stability with some localized offsets within the timeframe presented [36][37].

Storms in the GOA occur, but are typically limited to a few meters height and with similarly limited depth influence. When they occur, the nearshore and beach is heavily impacted, producing storm berms of up to a meter and higher, as coarser materials are lifted and concentrated and smaller grain sizes carried away. However, storms have not been observed to impact depths beyond a few meters, and do not produce currents that move suspended sediments far distances in short time scales.

The critical slope gradient for slumping or failure typically requires over 4–5° [38]. The maximum slope of the North Beach from the shoreline to 30 meters is only up to ~3°[39], and the distance between the position that the core was collected and the shoreline is about 300 meters. Slumping events and slope failures disassociated to canyons are characterized by scars in the region where the failure occurs, and a mound feature marking the primary depositional area. Such features are not present on the surface at the north beach study site, nor are they described in the subsurface [40]. Similarly, at the Tur Yam site, the slope at the coring location is also around ~3°, and there are no morphological indications for slumping or failure. Also, the Tur Yam anomalous deposits are heavier in coral debris, which is not present upslope, but rather in areas further south and in deeper water, suggesting their movement laterally and/or from deep to shallow rather than from shallower to deeper waters.

### The case for a Tsunamigenic Origin for Anomalous horizons

The recent increase in research on modern tsunami deposits such as the 2004 Indian Ocean Boxing Day, Chile 2010, and Tohoku, Japan 2011, has significantly expanded the comparative database against which paleotsunami deposits can be compared, identified and interpreted. Broadly defined, tsunami deposits are laterally continuous allochtonous deposits that cannot be accounted for by typical background transport processes (e.g. storms, floods, sea level, etc.) but do share characteristics with tsunamigenic indicators appropriate for their specific environment. Many of the characteristics of the anomalous horizons presented here have been described in other studies related to modern and paleo tsunami events. The following provides a discussion of these similarities and parallels.

Eroded, blackened, and yellowed foraminifer tests, which are present in the anomalous horizon, were described as a characteristic of the tsunami deposits left behind by the 2004 Sumatra earthquake [41] and the 2011 Tohoku Japan event [42]. Elsewhere it has been used as a possible indicator of older tsunami events or turbidites [43][44].

The character of the anomalous horizon in the North Beach core is indicative of a deposit laid by a transport mechanism capable of eroding, entraining, and transporting shallow uppershelf beach deposits and other nearshore terrestrial sediments, and depositing them at about 18m water depth. The deposit left behind was thick enough to survive later bioturbation, flooding, and storm events. Storm events are not known to be depositional offshore, flood events consist of distinctive fine sediments and tend to be depositional and not erosional, tectonics and sea-level have no association to these depths, leaving tsunami-related causes as a plausible explanation.

The massive coral and shell fragments present in the anomalous horizon of Tur Yam core are indicative of transported pieces based on their isolation, lack of living framework, older ages, and age reversal. Overall foraminifer maximum abundance in all horizons of Tur Yam core are greater than in the North Beach core, and while the abundance drops in the North Beach core within the anomalous horizon, the abundance in the Tur Yam horizon is relatively stable. The unique horizon does exhibit a three-fold increase in the ratio of larger to smaller foraminifera as well as eroded, yellowed, or blackened tests (see Fig 4). This is interpreted as the effect of selective transport and removal of the smaller test fraction particularly in the depositional phases. The overall consistency of the grain size distribution, combined with the observation that the difference in foraminifera abundance is not specific to the anomalous horizon suggest that the wave inundation inland may have been more limited at this location, and transport strength reduced, resulting in only the addition of entrained and transported older already loose coral fragments, while the matrix sediment was mostly entrained, selectively transported, and redeposited at a similar depth. While tsunamis cause coral damage, the presence of corals can mitigate tsunami damage [45][46][47], which could account for this intersite variation. Similar to the North Beach core, the Tur Yam core was collected beyond the depth in which sea-level change or tectonic displacement can be considered.

**Fig 7:**
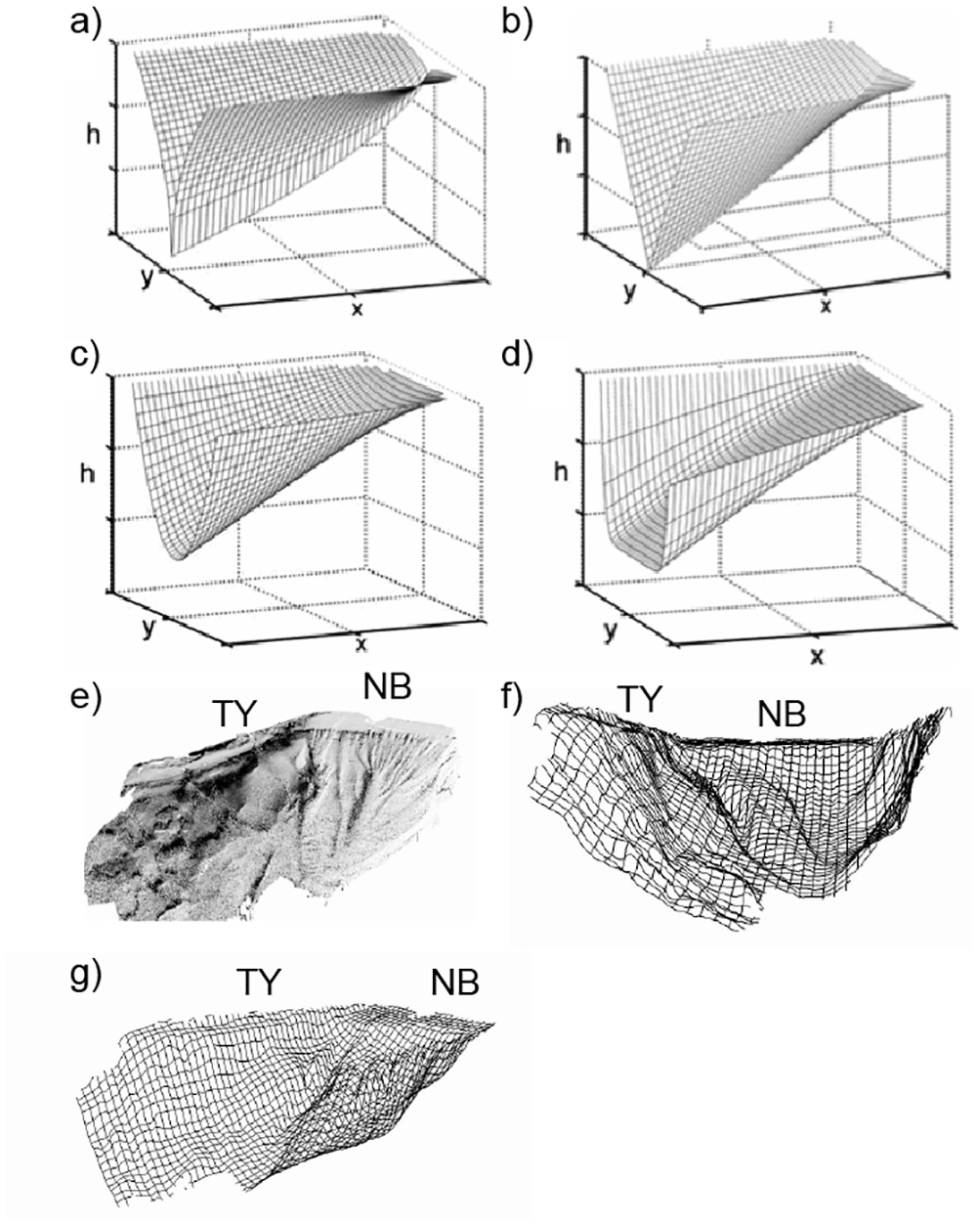
Three-dimensional mesh models. a-d) 3D mesh models of bottom morphologies conducive to abnormal amplification and run-up scenarios [50] compared to GOA e)shaded and f and g) mesh model maps [48]. NB = North Beach, TY = Tur Yam.

The presence of a thick deposit suggesting a synchronous event in both the northernmost and western side of the GOA suggests that the event had substantially more impact in terms of geographical range and inundation potential than the recently witnessed Nuweiba tsunami, which left no recognizable sedimentological indicators in the study’s cores, or the historically-recorded 1068 AD event, which may have left only a slight suggestion in the grain distribution values in the North Beach core (at approximately 40cm in North Beach core, Fig 4). The bathymetric geometry of the gulf [48][49] (Fig 2) resembling more closely a fjord than a sea, could lead to increased run-up and amplitude as a result of limited internal reflection en route to the shoreline [50] (Fig 7). The rapidly buried corals identified previously [17] provide important corroborating evidence for a possible tsunami and likely presents contemporaneous evidence of vertical tectonic offset. This earthquake may have caused seismically-induced mass wasting along the steep continental slope (Fig 2). The variations between the contemporaneous anomalous horizon from core to core also illustrates how the localized background sedimentological variations can result in distinctive and unique tsunami signatures, even within one geographical area. This observation is in agreement with others reported modern and ancient tsunami field studies [51][52][53][1][43][54]. The preservation of tsunami terrestrial and littoral deposits is highly dependent on environmental conditions (e.g. tropical versus desert, seasonality, presence of coastal lakes, etc.) population density and socioeconomic circumstances [55], search effort and design. In conditions such as those of the Andaman sea cost of Thailand, there was near-immediate alteration and erasure of terrestrial and coastal tsunamigenic deposits [56]. While preservation limitations also exist in the offshore submerged environment, thus far it is proving to be worthy of more thorough investigation in the effort towards discovering and studying tsunami deposits [24][57].

#### Chronological and historical context

The radiocarbon age from the North Beach places the maximum age at 100–400 BC (2 sigma error), while the Tur Yam radiocarbon age brackets the horizon as a minimum age of 100–500 BC (2 sigma error). Highest probability of these two radiocarbon ages place the event at about 2300 yBP, or around 200–300 BC (Data Repository). There is no preserved written record of a tsunami event recorded from that time and location. When the date is placed within the context of archaeological data, there seems to be a period of time without evidence for a settlement in the area. The archaeological site Tel el-Kheleifeh dates to the Iron Age period (~1200–1000 BC) through early 4^th^ century BC [58]. Later, an important Roman port site (Aila) that linked the region’s Nabatean cities to surrounding areas, was located on the Jordanian side of the GOA [5] [59]. A few hundred years between these phases present little activity, which might be related a population shift or economic failure and abandonment following the establishment of Berenike harbor by Ptolemy II (Philadelphos) in 275 BC further to the south [60].

## Conclusions

New GOA data suggests that at least one undocumented, possibly large, tsunami that occurred in the geologically recent past. This event was not identified before because the shallow offshore sediments have only recently begun to be explored. This hyperarid area is strategic economically and politically because Aqaba is Jordan’s only access to the sea, a flourishing tourism industry exists in Egypt, Israel and Jordan, and a rapidly growing residential population depends on key infrastructure (water, electricity, importation of food) to survive here. As there is yet no means to predict tsunamis, and a single tsunami of any significant size could have devastating consequences, it is advisable to take this into consideration in any development plans, risk assessments, and coastal management strategies. It is reasonable to assume that many of today’s populated coastlines are similarly underreported with regard to their past tsunami events due to a lack of historical records and efforts such as those presented here will help correct these gaps.

## Supporting Information

S1 File Table A: Chronological Report, Table B: North Beach radiocarbon calibration details, Table C: Tur Yam radiocarbon calibration details, Table D: North Beach foraminifera calculation data; Table E: Tur Yam foraminifera calculation data. (XLS)
